# Prevalence and risk factors associated with high-grade anal squamous intraepithelial lesions (HSIL)-AIN2 and HSIL-AIN3 in homosexual men

**DOI:** 10.1016/j.pvr.2016.05.003

**Published:** 2016-05-24

**Authors:** Dorothy A. Machalek, Fengyi Jin, I. Mary Poynten, Richard J. Hillman, David J. Templeton, Carmella Law, Jennifer M. Roberts, Sepehr N. Tabrizi, Suzanne M. Garland, Annabelle Farnsworth, Christopher K. Fairley, Andrew E. Grulich, on behalf of the SPANC study team

**Affiliations:** a HIV Epidemiology and Prevention Program, The Kirby Institute for infection and immunity in society, University of New South Wales, Sydney, Australia; b Department of Microbiology and Infectious Diseases, Royal Women's Hospital and Murdoch Childrens Research Institute, Melbourne, Australia; c Western Sydney Sexual Health Centre, Western Sydney Local Health District, Parramatta, New South Wales 2137, Australia; d Centre for Infectious Diseases and Microbiology, Westmead Clinical School, University of Sydney, New South Wales 2006, Australia; e St Vincent's Hospital, Sydney, New South Wales, Australia; f RPA Sexual Health, Sydney Local Health District, Sydney, Australia; g Douglass Hanly Moir Pathology, Sydney, New South Wales, Australia; h Department of Obstetrics and Gynaecology, University of Melbourne, Parkville, Victoria, Australia; i Melbourne Sexual Health Centre, Carlton, Victoria, Australia and Central Clinical School Monash University, Victoria, Australia

**Keywords:** AIN, anal intraepithelial neoplasia, ASIL, anal squamous intraepithelial lesion, CIN, cervical intraepithelial neoplasia, HSIL, high grade squamous intraepithelial lesion, HRA, high resolution anoscopy, HIV, human immunodeficiency virus, HPV, human papillomavirus, MSM, men who have sex with men, SPANC, Study for the Prevention of Anal Cancer, Risk factors, Surrogate endpoints, HSIL, Cancer screening, Human papillomavirus

## Abstract

**Background:**

Anal intraepithelial neoplasia grade 2 (AIN2) and AIN grade 3 (AIN3) are commonly grouped together as high grade squamous intraepithelial lesions (HSIL). We assessed risk factors for HSIL-AIN2 and HSIL-AIN3 in a cohort of homosexual men.

**Methods:**

At the baseline visit in the Study for the Prevention of Anal Cancer (SPANC), all men completed a questionnaire and underwent anal swabbing for cytology and HPV genotyping, followed by high resolution anoscopy.

**Results:**

Composite-HSIL prevalence was 47% and 32% among 220 HIV-positive and 396 HIV-negative men, respectively. HSIL-AIN3 (37.7% versus 24.7%; p<0.001), but not HSIL-AIN2 (9.5% versus 7.6%; p=0.395) was more common in HIV-positive men. Recent receptive anal partners (p-trend=0.045), and increasing number of high-risk (HR)-HPV types (p-trend<0.001) were associated with HSIL-AIN2. Lifetime receptive partners (p-trend<0.001), HIV status (OR 1.74; 95% CI: 1.05–2.87) and HPV16 (OR 3.00; 95% CI: 1.56–5.75) were associated with HSIL-AIN3. HPV16 was the most common HR-HPV type detected in men with HSIL-AIN3, both HIV-negative (61.1%) and HIV-positive (54.9%). HPV16 was less commonly detected in men with HSIL-AIN2.

**Conclusions:**

Grouping HSIL-AIN2 and HSIL-AIN3 as HSIL may mask considerable heterogeneity in anal cancer risk. Given the strong link between HPV16 and anal cancer, men with HSIL-AIN3 and HPV16 are likely to be at greatest risk of cancer.

## 1 Introduction

Population-based screening programs have been successful in reducing human papillomavirus (HPV)-associated cervical cancer incidence and mortality. As an understanding that HPV also causes anal squamous intraepithelial lesions (ASIL) and anal cancer [1], [2], [3] has emerged, some researchers have advocated a similar anal cancer screening program among the highest risk groups, namely men who have sex with men (MSM) and people with HIV [4].

Although anal and cervical high grade SIL (HSIL) share clinical and histological similarities [5], aspects of the epidemiology and natural history of the conditions in screened populations may be different. In particular, anal HSIL prevalence in MSM greatly exceeds that of cervical HSIL in women in the general population. In a recent meta-analysis, HSIL prevalence in MSM was 29.1% in HIV-positive, and 21.5% in HIV-negative men [6]. HSIL prevalence in MSM is so high that it is clear that most cases will never progress to anal cancer.

HSIL can be graded by the proportion of involvement of the epithelial layer as intraepithelial neoplasia grade 2 (IN2) or 3 (IN3) [7]. In the cervix, HSIL-cervical IN2 (HSIL-CIN2) is more likely to regress than HSIL-CIN3 [8]. However as the combined prevalence of CIN2 and CIN3 does not exceed 2% in a screening population [9] and both may progress to cancer, CIN2 and CIN3 are usually grouped together for treatment purposes [7]. The grouping of HSIL-AIN2 and HSIL-AIN3 as anal HSIL is also generally accepted for the clinical management. However, in contrast to the cervix, there have been no reported studies which have separately examined characteristics of HSIL-AIN2 and HSIL-AIN3.

We analysed baseline data from a predominantly community-recruited cohort of MSM in Sydney, Australia, to investigate risk factors for anal HSIL, and for HSIL-AIN 2 and HSIL-AIN3 separately.

## 2 Materials and methods

### 2.1 Study population

The Study of Prevention of ANal Cancer (SPANC) is an ongoing prospective study of HIV-positive and HIV-negative MSM, based in Sydney, Australia. Participants were recruited mainly from community-based settings. In addition, about 35% of HIV-positive and 5% of HIV-negative men were recruited through medical clinics. Eligible participants were men aged 35 years or over who reported sex with another man in their lifetime (any homosexual contact). Participants were excluded if they were unable to understand English, unable to attend scheduled follow-up visits or were unwilling to undergo high resolution anoscopy (HRA), had a bleeding disorder or were on anti-coagulation medications (except aspirin and other non-steroidal anti-inflammatory drugs). Men who had previously received HRA or those with a history of anal cancer were also excluded from the study [10]. The study was approved by the St Vincent's Hospital Human Research Ethics Committee (Sydney, Australia) and all participants gave written informed consent. The study protocol and the study's main objectives have been described in detail previously [10].

At the baseline visit, all men underwent anal swabbing for cytology and HPV genotyping immediately followed by high resolution anoscopy (HRA) and directed biopsy of any suspected HPV-associated abnormalities. Participants also completed detailed audio computer-assisted self-interviews (ACASI QDS, Bethesda, MD) [10], which collected information about potential risk factors such as lifetime and recent (in the previous six months) sexual practices and tobacco use. Additional information was sought from HIV-positive participants on CD4 T-lymphocyte count, HIV viral load and AIDS-defining illnesses.

### 2.2 HPV detection, anal cytology and HRA

The clinical and laboratory procedures performed in this study have been described in detail previously [10]. In brief, a saline-moistened Dacron swab was blindly inserted 3–5 cm into the anal canal and then gradually withdrawn while applying firm circumferential pressure to the wall of the anal canal for approximately one minute. The swab was then agitated in a vial containing PreservCyt (Hologic Corporation, Bedford, MA, USA). Prior to cytological processing, an aliquot of the medium was transferred to a separate tube for HPV testing. The remaining sample was used for cytological analysis. If the anal swab were deemed unsatisfactory for cytological evaluation, a repeat anal collection was performed approximately two weeks from the first. A ‘satisfactory’ slide was defined as having at least 2000 nucleated squamous cells.

The HRA was performed immediately after the anal swab [10]. Following insertion of a plastic anoscope and application of acetic acid, the anal canal was visualised under high-resolution magnification with further application of acetic acid (3% initially [10], but changed in January 2015 to 5% in response to evolving opinion that the higher concentration may allow better visualisation of SIL [11]), followed by Lugol's iodine, to help identify any HPV-associated abnormalities [11]. Abnormalities which were visually suggestive of ASIL were biopsied for histological assessment. Men who had no visual abnormalities did not undergo biopsy.

The samples were transferred to a pathology laboratory (Douglass Hanly Moir Pathology, Sydney, Australia) for processing and assessment. Reporting of cytology results was performed according to The Bethesda System TBS [12]. Reporting of biopsy results was performed in accordance with criteria, terminology and recommendations of the Lower Anogenital Squamous Terminology (LAST) Project [13], as described previously [10]. In particular, when a diagnosis of HSIL-AIN2 was proposed based on histomorphologic features on hematoxylin and eosin (H&E) stained slides, immunostaining for p16-INK4A (p16) was performed. Only strong uniform p16 immunostaining of the basal layer was considered positive. Positivity for p16 coupled with H&E features of AIN2 were both required for the diagnosis of HSIL-AIN2. If the p16 result was negative, the lesion was downgraded to LSIL or negative for SIL, depending on other criteria. Immunostaining for p16 was not performed in making diagnoses of straightforward LSIL or HSIL–AIN3. If multiple biopsies were taken, the result with the highest grade of disease was used for analysis [14].

HPV testing was performed on the anal PreservCyt specimens using the Roche LINEAR ARRAY (Roche Molecular Systems, Alameda, CA, US) to detect 37 individual HPV types with modifications as previously described [15]. As an in-house modification, samples that were negative for HPV and internal control were retested with half the volume of eluted DNA in order to reduce the inhibition due to high bacterial DNA content.

### 2.3 Statistical analysis

Given the acknowledged limitations in ASIL diagnosis of both histology and cytology [16] ASIL disease classification was based on composite endpoints of the most severe cytology or histology diagnoses, as outlined in Table 1. The exact binomial method was used to calculate 95% confidence intervals (CIs) for prevalence values. Odds ratios (ORs) and 95% CIs were estimated using logistic regression. Univariate and adjusted analyses were performed to identify risk factors for HSIL compared to men without ASIL, with further stratified analysis of risk factors for HSIL-AIN2 and HSIL-AIN3. Men in the composite-LSIL and ASC-H categories were excluded from this analysis. Sensitivity analyses restricted to histologically confirmed HSIL were also performed.

**Table 1:** Baseline prevalence of composite cytology-histology anal squamous intraepithelial lesions among 616 men in the Study of the Prevention of Anal Cancer, stratified by HIV status.

**ASIL category**	**Definition**	**All men (N=616)**		**HIV-Negative men (n=396)**		**HIV-Positive men (N=220)**		**p-Value**
		**n**	**% (95% CI)**	**n**	**% (95% CI)**	**n**	**% (95% CI)**	
**Composite-negative**	Visually normal HRA with no biopsy taken, or no evidence of ASIL on any biopsy or cytology	183	29.7 (26.2–33.4)	135	34.1 (29.6–38.9)	48	21.8 (16.8–27.8)	0.001
**Composite-LSIL**	LSIL or ASCUS cytology and/or LSIL histology as the highest grade of ASIL	155	25.2 (21.9–28.8)	97	24.5 (20.5–29.0)	58	26.4 (20.9–32.6)	0.609
**Composite-ASC-H**	ASC-H cytology and no HSIL (≤LSIL) on histology	46	7.5 (5.6–9.8)	36	9.1 (6.6–12.4)	10	4.5 (2.5–8.3)	0.040
**Composite-HSIL**	HSIL detected by either cytology or histology	232	37.7 (33.9–41.6)	128	32.3 (27.9–37.1)	104	47.3 (40.7–53.9)	<0.001
**Composite HSIL-AIN2**	HSIL-AIN2 on cytology and/or AIN2 on histology withoutAIN3 on either	51	8.3 (6.3–10.7)	30	7.6 (5.3–10.6)	21	9.5 (6.3–14.2)	0.395
**Composite HSIL-AIN3**	HSIL-AIN3 on cytology or histology	181	29.4 (2.6–33.1)	98	24.7 (20.7–29.3)	83	37.7 (31.5–44.4)	<0.001

Abbreviations: ASIL: Anal squamous intraepithelial lesion; HRA: High resolution anoscopy; AIN: Anal Intraepithelial neoplasia; ASC-US: Atypical squamous cells of undetermined significance; LSIL: low-grade squamous intraepithelial lesions; ASC-H: Atypical-squamous cells cannot exclude high-grade squamous intraepithelial lesion; HSIL: high-grade squamous intraepithelial lesion; HSIL-AIN2 and HSIL-AIN3; HPV: Human papillomavirus; HR-HPV: High-risk HPV.

For each of the above outcomes, we evaluated the following factors: age, HIV status, smoking exposure (never, past, current with ≤10 pack years of exposure, and current with >10 packs years of exposure), HPV16 status, number of HR-HPV types and the number of lifetime and recent (previous six months) receptive anal intercourse partners with and without a condom. Given the causal role of HPV in HSIL, the overall effect of sexual behaviours was first examined without considering HPV status. Sexual behaviour variables which were associated with each outcome at p<0.100 were considered in the initial multivariate model. Only variables that remained significant (p<0.05) were retained, along with age, HIV status and smoking in the final multivariate analyses (Table 3). In separate analyses we examined the association of increasing number of HR-HPV types with HSIL, HSIL-AIN2 and HSIL-AIN3 (Table 4) adjusting for HPV16, age, HIV status and smoking (Table 4, Stratum A). Given the known strong relationship between HPV 16 and anal cancer risk [2], we also conducted this analysis stratified by HPV16 status (Table 4, Stratum B and Stratum C). Among HIV-positive men, factors relating to HIV disease including, nadir and current CD4^+^ T-cell counts, HIV viral load, anti-retroviral treatment (ART) status, and history of AIDS-defining illness, were assessed individually adjusted for age, smoking status, HPV16 status and number of HR-HPV types (Table 5). Data analyses were performed using STATA version 14 (Stata Corporation, College Station, TX, US).

## 3 Results

Among the 617 participants enrolled from September 2010 to August 2015, 397 (64.3%) were HIV-negative and 220 (35.7%) were HIV-positive. The median age at enrolment was 49 years (IQR: 43–56), with no difference by HIV status (p=0.786). Nearly all men (95.3%) identified as gay or homosexual, 2.9% identified as bisexual and 1.1% identified as “other”. Four (0.7%) men identified as heterosexual. Among HIV-positive participants, the median time since their HIV diagnoses was 15 years (range: 0–30). Most (93.6%) were currently on ART, reported an undetectable HIV viral load (89.5%) and had a CD4 count of more than 350 cells/µl (88.0%).

Cytology data were available for all 617 participants. Among the HIV-negative, 22 (5.5%) men had repeatedly unsatisfactory anal swabs, 169 (42.6%) had no ASIL, 61 (15.4%) ASC-US, 19 (4.8%) LSIL, 60 (15.1%) ASC-H, 13 (3.3%) HSIL-AIN2 and 53 (13.4%) HSIL-AIN3. Among the HIV-positive, 7 (3.2%) men had repeatedly unsatisfactory anal swabs, 72 (32.7%) had no ASIL, 42 (19.1%) ASC-US, 28 (12.7%) LSIL, 28 (12.7%) ASC-H, 8 (3.6%) HSIL-AIN2 and 35 (15.9%) HSIL-AIN3. HRA data were available for 616 participants. One HIV-negative man was excluded because he did not tolerate HRA. Among the HIV-negative, 193 (48.7%) men had a visually normal HRA or had biopsies showing no evidence of ASIL, 100 (25.3%) LSIL, 29 (7.3%) HSIL-AIN2 and 74 (18.7%) HSIL-AIN3. Among the HIV-positive, 72 (32.7%) men had a visually normal HRA or had biopsies showing no evidence of ASIL, 55 (25.0%) LSIL, 20 (9.1%) HSIL-AIN2 and 73 (33.2%) HSIL-AIN3. One HIV-positive man who had a superficially invasive anal cancer arising from an HSIL lesion was classified as HSIL in these analyses. The baseline prevalence of composite-ASIL endpoints is presented in Table 1, stratified by HIV status. Hereafter, SIL outcomes referred to are composite endpoints unless otherwise specified.

In univariate analysis HSIL was significantly associated with being HIV-positive (p<0.001), having more lifetime receptive partners with (p-trend<0.001) and without a condom (p-trend<0.001), and having more receptive partners with (p=0.022) and without (p=0.004) a condom in the previous six months (Table 2). In stratified analysis, both HSIL-AIN2 and HSIL-AIN3 were significantly associated with HIV positivity (p=0.040 and p<0.001, respectively) and with having more lifetime receptive partners without a condom (p-trend<0.018 and p-trend<0.001, respectively). In addition, HSIL-AIN2 was significantly associated with having more receptive partners with (p-trend<0.001) and without (p-trend<0.001) a condom in the previous six months (Table 2).

**Table 2:** Univariate analysis of risk factors for prevalent composite-HSIL in the Study of the Prevention of Anal Cancer, overall and stratified by composite-AIN2 and composite-AIN3 diagnoses.

		**Composite-HSIL versus composite-negative**^a^**(n=415)**			**Composite-AIN2 versus composite-negative**^b^**(n=234)**			**Composite-AIN3 versus Composite-negative**^c^**(n=364)**		
**Variable**		**n (%)**	**OR (95% CI)**	**p-Value**	**n (%)**	**OR (95% CI)**	**p-Value**	**n (%)**	**OR (95% CI)**	**p-Value**
**Age**	**35–44 years**	74/131 (56.5)	1.00	0.577	21/78 (26.9)	1.00	0.113	53/110 (48.2)	1.00	0.991
	**45–54 years**	89/161 (55.3)	0.95 (0.60–1.52)		18/90 (20.0)	0.68 (0.33–1.40)		71/143 (49.7)	1.06 (0.64–1.75)	
	**55–64 years**	52/84 (61.6)	1.25 (0.71–2.20)		10/42 (23.8)	0.85 (0.35–2.03)		42/74 (56.8)	1.41 (0.78–2.56)	
	**65+ years**	17/39 (43.6)	0.60 (0.29–1.23)		2/24 (8.3)	0.25 (0.05–1.18)		15/37 (40.5)	0.73 (0.34–1.57)	
**HIV status**	**HIV-negative**	128/263 (48.7)	1.00	**<0.001**^**1**^	30/165 (18.2)	1.00	**0.040**^**1**^	98/233 (42.1)	1.00	**<0.001**^**1**^
	**HIV-positive**	104/152 (68.4)	2.29 (1.50–3.47)		21/69 (30.4)	1.97 (1.03–3.76)		83/131 (63.4)	2.38 (1.53–3.70)	
										
**Smoking status**	**Never**	120/223 (53.8)	1.00	0.104	26/129 (20.2)	1.00	0.586	94/197 (47.7)	1.00	0.084
	**Past**	76/138 (55.1)	1.05 (0.69–1.61)		18/80 (22.5)	1.15 (0.58–2.25)		58/120 (48.3)	1.03 (0.65–1.61)	
	**Current ≤10PY**	12/19 (63.2)	1.47 (0.56–3.88)		5/12 (41.7)	2.83 (0.83–9.64)		7/14 (50.0)	1.10 (0.37–3.24)	
	**Current >10PY**	24/35 (68.6)	1.87 (0.88–4.01)		2/13 (15.4)	0.72 (0.15–3.45)		22/33 (66.7)	2.19 (1.01–4.76)	
**HPV16 status**	**Negative**	114/273 (41.8)	1.00	**<0.001**^1^	40/199 (20.1)	1.00	0.093^1^	74/233 (31.8)	1.00	**<0.001**^1^
	**Positive**	114/136 (83.8)	7.23 (4.31–12.11)		11/33 (33.3)	1.99 (0.89–4.43)		103/125 (82.4)	10.06 (5.88–17.20)	
										
**Number of HR-HPV types**	**0**	25/134 (18.7)	1.00	**<0.001**	9/118 (7.6)	1.00	**<0.001**	16/125 (12.8)	1.00	**<0.001**
	**1**	55/98 (56.1)	5.58 (3.09–10.06)		10/53 (18.9)	2.82 (1.07–7.41)		45/88 (51.1)	7.13 (3.65–13.94)	
	**2**	66/84 (78.6)	16.00 (8.11–31.51)		17/35 (48.6)	11.44 (4.43–29.56)		49/67 (73.1)	18.55 (8.73–39.38)	
	**3+**	82/93 (88.2)	32.50 (15.16–69.83)		15/26 (57.7)	16.52 (5.88–46.41)		67/78 (85.9)	41.49 (18.17–94.76)	
										
**Lifetime receptive partners with a condom**	**0–1**	5/24 (20.8)	1.00	**<0.001**	0/19		**<0.001**	5/24 (20.8)	1.00	**<0.001**
	**2–5**	19/52 (36.5)	2.19 (0.69–6.96)		2/35 (5.7)	N/A		17/50 (34.0)	1.96 (0.61–6.27)	
	**6–10**	25/42 (59.5)	5.59 (1.58–19.75)		4/21 (19.1)	N/A		21/38 (55.3)	4.69 (1.33–16.53)	
	**>10**	176/286 (61.5)	6.08 (2.15–17.22)		45/155 (29.0)	N/A		131/241 (54.4)	4.53 (1.60–12.79)	
										
**Lifetime receptive partners without a condom**	**0–1**	18/63 (28.6)	1.00	**<0.001**	4/49 (8.2)	1.00	**0.018**	14/59 (23.7)	1.00	**<0.001**
	**2–5**	52/105 (49.5)	2.45 (1.24–4.86)		18/71 (25.4)	3.82 (1.17–12.53)		34/87 (39.1)	2.06 (0.97–4.37	
	**6–10**	56/85 (65.9)	4.83 (2.25–10.36)		5/34 (14.7)	1.94 (0.47–7.95)		51/80 (63.8)	5.65 (2.48–12.88)	
	**>10**	106/162 (65.4)	4.73 (2.41–9.30)		24/80 (30.0)	4.82 (1.49–15.57)		82/138 (59.4)	4.71 (2.27–9.78)	
										
**Previous six months, receptive partners with a condom**	**None**	91/180 (50.6)	1.00	**0.022**	13/102 (12.8)	1.00	**<0.001**	78/167 (46.7)	1.00	0.194
	**One**	40/70 (57.1)	1.30 (0.75–2.28)		8/38 (21.1)	1.83 (0.68–4.87)		32/62 (51.6)	1.22 (0.68–2.19)	
	**2–5**	53/94 (56.4)	1.26 (0.76–2.09)		15/56 (26.8)	2.50 (1.08–5.83)		38/79 (48.1)	1.06 (0.62–1.81)	
	**>5**	48/71 (67.6)	2.04 (1.14–3.66)		15/38 (39.5)	4.46 (1.78–11.17)		33/56 (58.9)	1.64 (0.88–3.04)	
										
**Previous six months, receptive partners without a condom**	**None**	103/197 (52.3)	1.00	**0.004**	16/110 (14.6)	1.00	**<0.001**	87/181 (48.1)	1.00	0.054
	**One**	62/121 (51.2)	0.96 (0.61–1.51)		14/73 (19.2)	1.39 (0.63–3.08)		48/107 (44.9)	0.88 (0.54–1.42)	
	**2–5**	39/62 (62.9)	1.55 (0.86–2.79)		12/35 (34.3)	3.07 (1.25–7.54)		27/50 (54.0)	1.27 (0.68–2.38)	
	**>5**	28/35 (80.0)	3.65 (1.49–8.93)		9/16 (56.3)	7.55 (2.27–25.09)		19/26 (73.1)	2.93 (1.16–7.42)	

Numbers do not always add up to column totals because of small amounts of missing data.

PY: Pack Years.

^1^ Score test of homogeneity, else the p-values presented are score test for trend of odds.

^a^ Comparing men with composite negative result (n=183) to men with HSIL detected on by either cytology or histology (n=232).

^b^ Comparing men with composite negative result (n=183) to men with HSIL-AIN2 on cytology and/or AIN2 on histology without a diagnosis of AIN3 on either (n=51).

^c^ Comparing men with composite negative result (n=183) to men with HSIL-AIN3 on cytology or histology (n=181).

Men in the composite-LSIL (n=155) and composite-ASC-H (n=55) were excluded from analyses of risk factors.

Row percentages presented.

In multivariate analyses (Table 3) behavioural risk factors that were independently associated with HSIL included HIV positivity (p=0.035), and having more lifetime receptive partners with (p-trend=0.032) and without (p-trend=0.001) a condom. Factors that were independently associated with HSIL-AIN2 included receptive practices with (p-trend=0.044) and without (p-trend=0.045) a condom in the previous six months. For HSIL-AIN3, HIV positivity (p=0.031) and having more lifetime receptive partners without a condom (p<0.001) were significantly associated (Table 3).

**Table 3:** Multivariate analysis of sexual behaviour risk factors for prevalent composite-HSIL in the Study of the Prevention of Anal Cancer, overall and stratified by composite-AIN2 and composite-AIN3 diagnoses.

		**Composite-HSIL versus composite-negative**^a^**(n=415)**			**Composite-AIN2 versus composite-negative**^b^**(n=234)**			**Composite-AIN3 versus Composite-negative**^c^**(n=364)**		
**Variable**		**n (%)**	**Adjusted OR (95% CI)**	**p-Value**	**n (%)**	**Adjusted OR (95% CI)**	**p-Value**	**n (%)**	**Adjusted OR (95% CI)**	**p-Value**
**HIV status**	**HIV-negative**	128/263 (48.7)	1.00	**0.035**^1^	30/165 (18.2)	1.00	0.436^1^	98/233 (42.1)	1.00	**0.031**^1^
	**HIV-positive**	104/152 (68.4)	1.69(1.03–2.76)		21/69 (30.4)	1.35 (0.64–2.85)		83/131 (63.4)	1.74 (1.05–2.87)	
										
**Lifetime receptive partners with a condom**	**0–1**	5/24 (20.8)	1.00	**0.032**						
	**2–5**	19/52 (36.5)	1.79 (0.66–4.87)							
	**6–10**	25/42 (59.5)	2.24 (0.82–6.11)							
	**>10**	176/286 (61.5)	2.51 (1.03–6.08)							
										
**Lifetime receptive partners without a condom**	**0–1**	18/63 (28.6)	1.00	**0.001**				14/59 (23.7)	1.00	**<0.001**
	**2–5**	52/105 (49.5)	2.24 (1.13–4.42)					34/87 (39.1)	2.08 (0.99–4.36)	
	**6–10**	56/85 (65.9)	3.92 (1.90–8.12)					51/80 (63.8)	5.07 (2.37–10.85)	
	**>10**	106/162 (65.4)	3.26 (1.62–6.56)					82/138 (59.4)	3.61 (1.74–7.49)	
										
**Previous six months, receptive partners with a condom**	**None**				13/102 (12.8)	1.00	**0.044**			
	**One**				8/38 (21.1)	1.67 (0.62–4.52)				
	**2–5**				15/56 (26.8)	2.06 (0.84–5.00)				
	**>5**				15/38 (39.5)	2.66 (0.96–7.39)				
										
**Previous six months, receptive partners without a condom**	**None**				16/110 (14.6)	1.00	**0.045**			
	**One**				14/73 (19.2)	1.26 (0.56–2.82)				
	**2–5**				12/35 (34.3)	1.84 (0.66–5.08)				
	**>5**				9/16 (56.3)	4.16 (1.13–15.27)				

^1^ Score test of homogeneity, else the p-values presented are score test for trend of odds.

^a^ Comparing men with composite negative result (n=183) to men with HSIL detected on by either cytology or histology (n=232).

^b^ Comparing men with composite negative result (n=183) to men with HSIL-AIN2 on cytology and/or AIN2 on histology without a diagnosis of AIN3 on either (n=51).

^c^ Comparing men with composite negative result (n=183) to men with HSIL-AIN3 on cytology or histology (n=181).

Men in the composite-LSIL (n=155) and composite-ASC-H (n=55) were excluded from analyses of risk factors.

Adjusted for age, smoking exposure and the variables presented in the table.

In separate analyses examining the association of HSIL, HSIL-AIN2 and HSIL-AIN3 with increasing number of HR-HPV types (Table 4 Stratum A), HSIL was strongly associated with increasing number of HR-HPV types (p-trend<0.001) and HPV16 positivity (p=0.028). This was also the case for HSIL-AIN2 and HSIL-AIN3 examined separately (p-trend<0.001 for each) (Table 4 Stratum A). HSIL-AIN3 was strongly associated with HPV16 positivity (p<0.001). In contrast, HSIL-AIN2 had a negative association with HPV16 (OR; 0.35, 95% CI: 0.12–1.00; p=0.050).

**Table 4:** Association of the number of HR-HPV with prevalent composite-HSIL, among HR-HPV positive men, in the Study of the Prevention of Anal Cancer, overall and stratified by composite-AIN2 and composite-AIN3 diagnoses.

		**Composite-HSIL versus composite-negative**^a^**(n=415)**			**Composite-AIN2 versus composite-negative**^b^**(n=234)**			**Composite-AIN3 versus Composite-negative**^c^**(n=364)**		
**Variable**		**n (%)**	**Adjusted OR (95% CI)**	**p-Value**	**n (%)**	**Adjusted OR (95% CI)**	**p-Value**	**n (%)**	**Adjusted OR (95% CI)**	**p-Value**
**Stratum A: Among all HR-HPV positive men**										
**HPV16**	**Negative**	89/13 (64.0)	1.00	**0.028**^1^	31/81 (38.3)	1.00	0.050^1^	58/108 (53.7)	1.00	**0.001**^1^
	**Positive**	114/136 (83.8)	2.02 (1.08–3.79)		11/33 (33.3)	0.35 (0.12–1.00)		103/125 (82.4)	2.91 (1.52–5.57)	
										
**Number of HR-HPV types**	**1**	55/98 (56.1)	1.00	<0.001	10/53 (18.9)	1.00	<0.001	45/88 (51.1)	1.00	<0.001
	**2**	66/84 (78.6)	2.70 (1.39–5.26)		17/35 (48.6)	4.85 (1.80–13.09)		49/67 (73.1)	2.23 (1.10–4.53)	
	**3+**	82/93 (88.2)	4.30 (1.94–9.53)		15/26 (57.5)	10.23 (3.04–34.43)		67/78 (85.9)	3.58 (1.57–8.14)	

**Stratum B: Among HPV16 positive men**										
**Number of HR-HPV types**	**1**	23/30 (76.7)	1.00	0.071	1/8 (12.5)	1.00	**0.033**	22/29 (75.9)	1.00	0.111
	**2**	27/35 (77.1)	1.01 (0.33–3.28)		2/10 (20.0)	2.16 (0.15–30.51)		25/33 (75.8)	0.96 (0.29–3.14)	
	**3+**	64/71 (90.0)	2.73 (0.83–8.92)		8/15 (53.0)	10.44 (0.93–117.13)		56/63 (88.9)	2.43 (0.74–7.99)	

**Stratum C: Among HPV16 negative but other HR-HPV positive men**										
**Number of HR-HPV types**	**1**	32/68 (47.1)	1.00	**<0.001**	9/45 (20.0)	1.00	**0.001**	23/59 (39.0)	1.00	**0.004**
	**2**	39/49 (79.6)	4.35 (0.87–10.11)		15/25 (60.0)	5.97 (2.02–17.68)		24/34 (70.6)	3.62 (1.46–8.99)	
	**3+**	18/22 (81.8)	4.98 (1.52–16.32)		7/11 (63.4)	7.26 (1.71–30.80)		11/15 (73.3)	4.19 (1.19–14.81)	

All analyses presented were adjusted for HIV status, age, smoking exposure and the variables presented in the table.

1 Score test of homogeneity, else the p-values presented are score test for trend of odds.

a Comparing men with composite negative result to men with HSIL detected on by either cytology or histology.

b Comparing men with composite negative result to men with HSIL-AIN2 on cytology and/or AIN2 on histology without a diagnosis of AIN3 on either.

c Comparing men with composite negative result to men with HSIL-AIN3 on cytology or histology; Men in the composite-LSIL (n=155) and composite-ASC-H (n=55) were excluded from analyses of risk factors.

In analyses stratified by HPV16 status, when the analysis was limited to HPV16 positive men, HSIL-AIN2, but not HSIL-AIN3, remained significantly associated with increasing number of HR-HPV types (p-trend=0.033) (Table 4, Stratum B). Among HPV16 negative (other HR-HPV positive) men, both HSIL-AIN2 and HSIL-AIN3 remained strongly associated with increasing number of HR-HPV types (p-trend=0.001 and p-trend=0.004, respectively) (Table 4, Stratum C). HPV16 was the most commonly detected HR-HPV type detected in men with HSIL-AIN3, both in HIV-negative (61.1%) and HIV-positive (54.9%) (p=0.401) men. In contrast, HPV16 was less commonly detected in men with HSIL-AIN2, whether they were HIV-negative (26.7%) or HIV-positive (14.3%) (p=0.286).

Among HIV-positive men, neither HSIL-AIN2 nor HSIL-AIN3 outcomes were significantly associated with most recent CD4^+^ T-cell count, HIV viral load, ART treatment status or a history of AIDS defining illness. A non-significant trend was observed between HSIL-AIN3 and lower nadir CD4^+^ T-cell count (p-trend=0.080) (Table 5).

**Table 5:** Multivariate analysis of HIV factors associated with prevalent composite-HSIL among HIV-positive men in the Study of the Prevention of Anal Cancer, overall and stratified by composite-AIN2 and composite-AIN3 diagnoses.

		**Composite-negative versus Composite-HSIL**^a^**(n=152)**			**Composite-negative versus Composite-AIN2**^b^**(n=69)**			**Composite-negative versus Composite-AIN3**^c^**(n=131)**		
**Variable**		**n (%)**	**Adjusted OR (95% CI)**	**p-Value**	**n (%)**	**Adjusted OR (95% CI)**	**p-Value**	**n (%)**	**Adjusted OR (95% CI)**	**p-Value**
**Nadir CD4**^**+**^**T-cell count**	**≥500**	12/20 (60.0)	1.00	0.107	3/11 (27.3)	1.00	0.592	9/17 (52.9)	1.00	0.080
	**200–500**	38/61 (62.3)	1.10 (0.39–3.10)		9/32 (28.3)	1.04 (0.21–5.32)		29/52 (55.8)	1.12 (0.37–3.36)	
	**<200**	51/68 (75.0)	2.00 (0.70–5.71)		9/26 (34.6)	1.41 (0.30–6.67)		42/59 (71.2)	2.20 (0.72–6.64)	
										
**Result of last CD4**^**+**^**T-cell count**	**>500**	57/94 (60.6)	1.00	0.181	10/47 (21.3)	1.00	0.324	47/84 (56.0	1.00	0.248
	**500–351**	29/35 (82.9)	2.95 (1.02–8.52)		8/14 (57.1)	4.12 (1.05–16.01)		21/27 (77.8)	3.08 (0.98–9.69)	
	**≤350**	11/15 (73.3)	1.70 (0.43–6.74)		3/7 (42.9)	2.68 (0.44–16.14)		8/12 (66.7)	1.05 (0.20–5.52)	
										
**Result of last HIV viral load test**^d^	**Undetectable**	90/132 (68.2)	1.00	0.669	17/59 (28.8)	1.00	0.216	73/115 (63.5)	1.00	0.695
	**Detectable**	9/12 (75.0)	1.40 (0.30–6.63)		3/6 (50.0)	3.21 (0.51–20.38)		6/9 (66.7)	0.70 (0.12–4.18)	
										
**Currently on any ART**	**No**	5/7 (71.4)	1.00	0.754	2/4 (50.0)	1.00	0.373	3/5 (60.0)	1.00	0.666
	**Yes**	99/145 (68.3)	0.74 (0.12–4.72)		19/65 (29.2)	0.37 (0.04–3.30)		80/126 (63.5)	1.56 (0.21–11.68)	
										
**History of AIDS-defining illness**	**No**	66/102 (64.7)	1.00	0.319	16/52 (30.8)	1.00	0.768	50/86 (58.1)	1.00	0.175
	**Yes**	36/48 (75.0)	1.55 (0.65–3.70)		5/17 (29.4)	0.82 (0.22–3.03)		31/43 (72.1)	1.89 (0.75–4.71)	

^a^ Comparing men with composite negative result (n=48) to men with HSIL detected on by either cytology or histology (n=104).

^b^ Comparing men with composite negative result (n=48) to men with HSIL-AIN2 on cytology and/or AIN2 on histology without a diagnosis of AIN3 on either (n=21).

^c^ Comparing men with composite negative result (n=48) to men with HSIL-AIN3 on cytology or histology (n=83). Adjusted for number of HR-HPV types and HPV16 detected on anal swab, age and smoking exposure.

^d^ Undetectable viral load: <50 copies; Detectable viral load ≥50 copies.

Men in the composite-LSIL (n=155) and composite-ASC-H (n=55) were excluded from analyses of risk factors.

## 4 Discussion

In this cohort of HIV-positive and HIV-negative homosexual men, the baseline prevalence of composite-HSIL was 47% and 32%, respectively. Prevalence of HSIL-AIN3, but not HSIL-AIN2, was significantly higher in HIV-positive men. Significant predictors of composite HSIL-AIN2 included higher numbers of recent (but not lifetime) receptive anal intercourse partners, and increasing number of HR-HPV types. In contrast, predictors of composite HSIL-AIN3 included higher numbers of lifetime (but not recent) receptive anal intercourse partners, HIV status and HPV16 detection. These data suggest that risk of HSIL-AIN2 is associated with recent sexual behaviour, while HSIL-AIN3 is associated with HIV positivity and lifetime sexual exposures suggesting chronic, longstanding HR-HPV infection, particularly with HPV16. Given the strong link between HPV16 and anal cancer, men with HSIL-AIN3 and HPV16 are likely to be at greatest risk of anal cancer.

Substantial research has been reported on the relationship between HPV infection and HSIL-CIN2 and HSIL-CIN3 in women. HPV infection is most common in young women, who have higher number of recent sexual partners [17]. The time between incident HPV infection and appearance of HSIL can be short. HSIL has been diagnosed within two years of sexual debut with no difference by HSIL grade (HSIL-CIN2 or HSIL-CIN3) [18], [19], [20]. In young, sexually active women, approximately 40% of HSIL-CIN2 lesions will resolve without treatment, and HSIL-CIN2 caused by HR-HPV types other than HPV16 is particularly likely to regress [8]. Our results suggest that HSIL-AIN2 in homosexual men is similarly related to recent sexual behaviour and infection with HR-HPV types other than HPV16. Many homosexual men continue to have multiple sexual partners well into older age, and hence are likely to have higher rates of newly acquired HPV infections and of HSIL related to recent infection [21], [22].

The proportion of histological HSIL cases which were HSIL-AIN2 in SPANC, of 8% [16], was somewhat lower than the 13–35% reported in recent studies [23], [24], [25]. We believe that this may reflect the strict use of the LAST diagnostic criteria in our study. We stained all possible HSIL-AIN2 diagnoses with p16, and downgraded (to LSIL or negative for LSIL) those cases which did not have strong uniform staining of the basal layer. We believe it is critical that studies in the field state precisely whether LAST guidelines broadly, and those regarding p16 staining specifically, have been used. Otherwise, direct comparison between studies is difficult.

The majority of HSIL diagnosed (78%) were HSIL-AIN3. HSIL-AIN3 was associated with lifetime sexual risk, suggesting chronic, longstanding HR-HPV infection. In the cervix, rates of progression to cancer are higher for HSIL-CIN3 than for HSIL-CIN2 [7]. Among HSIL-CIN3 lesions, there is substantial heterogeneity in risk of progressing to cervical cancer depending on the causative HR-HPV type [15]. HPV16 is clearly the type with the highest carcinogenic potential [26]. According to a recent global review, infection with HPV16 accounts for 87% of HPV positive invasive anal canal cancers worldwide [3]. In our study, HPV16 was detected in 58% of men with HSIL-AIN3. HPV16 was a strong predictor of composite HSIL-AIN3 and this was independent of the presence of other HR-HPV types. In view of the fact that the large majority of anal cancers are caused by HPV16, men with HPV16 and HSIL-AIN3 are likely to have a higher risk of anal cancer compared to those with HSIL-AIN3 associated with other HR-HPV types.

We found a strong association between number of HR-HPV types and risk of HSIL-AIN2 and HSIL-AIN3, consistent with a previous report of risk factors for composite-HSIL [25], and this remained strongly significant even after adjusting for the presence of HPV16. However, we found divergent results when we performed an analysis stratified by the presence of HPV16. Among HPV16 negative men, we found that increasing number of HR-HPV types was strongly associated with HSIL-AIN2 and HSIL-AIN3 risk. On the other hand, among HPV16 positive men, we found increasing number of HR-HPV types was associated with HSIL-AIN2, but not HSIL-AIN3 risk. This may indicate that HSIL-AIN2 is driven largely by non-HPV16 infections, whereas HSIL-AIN3 is most commonly due to HPV16. The absence of a significant effect of increasing number of HPV types in HPV16 positive men argues against a biological effect of interaction between HPV types on HSIL-AIN3 risk as has been similarly demonstrated in the cervix [27], [28].

We found that HIV-positive men were at higher risk of HSIL-AIN3, but not of HSIL-AIN2. MSM with HIV have a much higher risk of anal cancer than HIV negative MSM [6], [29], and our finding of higher HSIL-AIN3 is entirely consistent with this pattern. In our study decreasing nadir and current CD4^+^ T-cell count were not significantly associated with prevalent HSIL-AIN3. A significant relationship has been found by others [30], [31].

This study has several limitations. First, these baseline data are cross-sectional, so our ability to draw conclusions about the natural history of HSIL-AIN2 and HSIL-AIN3 is limited. However SPANC is a three year prospective study, and future reports will allow such longitudinal analyses. Second, the small sample size of HIV-positive men limits the statistical power of the analysis to detect potential associations between makers of immune function. Third, HPV testing was based on ThinPrep aliquot collected prior to cytological processing, and not on HPV detected within individual HSIL lesions. We did not detect HR-HPV in ThinPrep residuum in 9 (17.7%) men with HSIL-AIN2 and 16 (9.0%) men with HSIL-AIN3. There are three likely explanations for this given our use of composite cytology and histology endpoints. First, p16 staining was used to confirm histological but not cytological HSIL-AIN2. Among men with HSIL-AIN2 on cytology only, misclassification of a benign cytological mimic, specifically immature metaplasia as HSIL is possible [32], [33]. Second, among men with HSIL on histology only, the anal swab may have missed an HSIL lesion during sampling, therefore missing the associated (causative) HPV type. Last, infection with LR-HPV types may account for small proportion of HSIL. LR-HPV types particularly HPV6 and HPV11 have been associated with a small proportion of anal cancers [34].

A strength of the study was that recruitment was mostly community-based and likely to be representative of the target screening population of HIV-positive and HIV-negative MSM. The great majority identified as homosexual or gay. In addition, biopsy reporting was performed in accordance with the LAST Project [13], limiting potential misclassification of histological HSIL. Also, there was a very high degree of inter-rater reliability and intra-rater repeatability in histological diagnosis in the study [35]. Finally, the limited sensitivity of anal cytology and HRA to detect HSIL was addressed by using a composite cytology-histology endpoint. While this is a more accurate measure of disease burden, we recognise that histologically confirmed HSIL is ultimately the clinical outcome of interest. Sensitivity analyses were performed repeating the risk factor and HPV analyses comparing men with histological-AIN2 and histological-AIN3 to those who were ASIL negative, this did not affect the main study findings (data not shown).

In summary, we found an extremely high prevalence of HSIL in homosexual men, about four-fifths of which was the higher grade lesion, HSIL-AIN3. Of these, just over half (58%) had detectable HPV16, comprising 17% of all men enrolled in the study. While the combination of HSIL-AIN2 and HSIL-AIN3 as HSIL may be required for pathology reporting for clinical management purposes, in homosexual men it probably masks considerable heterogeneity in true anal cancer risk. Given the fact that HPV16 causes the great majority of anal cancer and the widespread availability of HPV genotyping assays, incorporation of testing for HPV16 in men with HSIL may identify those at highest risk of anal cancer. Large, high-quality prospective studies are required to inform whether HPV16 status is a useful marker of anal cancer risk.

## Conflict of interest statement

R. J Hillman has received support from CSL Biotherapies and MSD; S. M. Garland have received advisory board fees and grant support from CSL and GlaxoSmithKline, and lecture fees from Merck, GSK and Sanofi Pasteur; in addition, has received funding through her institution to conduct HPV vaccine studies for MSD and GSK and is a member of the Merck Global Advisory Board as well as the Merck Scientific Advisory Committee for HPV; J.M. Roberts and A. Farnsworth have received material supplies for research from Hologic (Australia) Pty Ltd; C.K. Fairley owns shares in CSL that makes Gardasil in Australia; A.E. Grulich has received honoraria and travel funding from Merck. All other authors have no conflicts of interest to declare.

## Source of financial support

This work was supported by the National Health and Medical Research Council Program Grant (Sexually transmitted infections: Causes, consequences and interventions Grant #568971); and a Cancer Council New South Wales Strategic Research Partnership Program Grant (Preventing morbidity and mortality from anal cancer Grant #13-11). Cytological testing materials were provided by Hologic (Australia) Pty Ltd. The Kirby Institute is affiliated with the Faculty of Medicine, University of New South Wales and funded by the Australian Government Department of Health and Ageing. The views expressed in this publication do not necessarily represent the position of the Australian Government.
